# Inhibition of oxidative phosphorylation suppresses the development of osimertinib resistance in a preclinical model of EGFR-driven lung adenocarcinoma

**DOI:** 10.18632/oncotarget.13388

**Published:** 2016-11-16

**Authors:** Matthew J. Martin, Cath Eberlein, Molly Taylor, Susan Ashton, David Robinson, Darren Cross

**Affiliations:** ^1^ AstraZeneca Oncology, Innovative Medicines, Alderley Park, Macclesfield, Cheshire, United Kingdom; ^2^ AstraZeneca Oncology, Innovative Medicines, CRUK Cambridge Institute, Cambridge, United Kingdom

**Keywords:** non-small cell lung cancer, signal transduction, drug resistance, metabolism, pre-clinical models of drug efficacy

## Abstract

Metabolic plasticity is an emerging hallmark of cancer, and increased glycolysis is often observed in transformed cells. Small molecule inhibitors that target driver oncogenes can potentially inhibit the glycolytic pathway. Osimertinib (AZD9291) is a novel EGFR tyrosine kinase inhibitor (TKI) that is potent and selective for sensitising (EGFRm) and T790M resistance mutations. Clinical studies have shown osimertinib to be efficacious in patients with EGFRm/ T790M advanced NSCLC who have progressed after EGFR-TKI treatment. However experience with targeted therapies suggests that acquired resistance may emerge. Thus there is a need to characterize resistance mechanisms and to devise ways to prevent, delay or overcome osimertinib resistance. We show here that osimertinib suppresses glycolysis in parental EGFR-mutant lung adenocarcinoma lines, but has not in osimertinib-resistant cell lines. Critically, we show osimertinib treatment induces a strict dependence on mitochondrial oxidative phosphorylation (OxPhos), as OxPhos inhibitors significantly delay the long-term development of osimertinib resistance in osimertinib-sensitive lines. Accordingly, growth conditions which promote a less glycolytic phenotype confer a degree of osimertinib resistance. Our data support a model in which the combination of osimertinib and OxPhos inhibitors can delay or prevent resistance in osimertinib-naïve tumour cells, and represents a novel strategy that warrants further pre-clinical investigation.

## INTRODUCTION

Activating mutations in the epidermal growth factor receptor (EGFR) have been reported in up to 15% and 40% of cases of non-small cell lung cancer (NSCLC) in Western and Asian populations, respectively [1]. This has prompted development of specific EGFR small molecule tyrosine kinase inhibitors, such as gefitinib and erlotinib that have shown significant clinical benefit in this segment. Unfortunately in a large majority of patients the response to TKIs is not sustained, with median relapse occurring 9–14 months after initiation of treatment [2, 3]. In approximately 60% of patients, resistance to these first-generation TKIs is due to a secondary T790M mutation in EGFR within the kinase domain which prevents drug binding [4–6]. Therefore strategies to target T790M EGFR were developed, and osimertinib (AZD9291; TAGRISSO^™^) was developed as a new class of TKI to target both T790M and activating-mutant forms of EGFR while showing lower potency against wildtype EGFR [7, 8]. Data from clinical trials have shown osimertinib to have a 66% response rate against NSCLC tumours that have become resistant to TKI treatment due to the T790M mutation [9], and has been approved by the US Food and Drug Administration and the European Commission for use in T790M-positive NSCLC patients who have progressed on EGFR TKI therapy. However it is anticipated that osimertinib resistance will arise after initial response. Emerging clinical data on osimertinib resistance has shown that a subset of patients acquire yet another EGFR mutation, C797S which also prevents drug binding [10], or can use bypass signaling [11]. However a majority of patients likely develop resistance by as yet unknown mechanisms, highlighting the need to develop alternative strategies to prevent progression after osimertinib treatment.

An emerging hallmark of cancer cells is altered metabolism, to adapt to the increased energy and biomass requirements that accompany transformation [12]. Notably, most cancer types exhibit enhanced glycolysis, a phenomenon known as the Warburg effect [13]. However rather than simply a mechanism to generate additional energy via ATP production, it is now thought that increased glycolysis in cancer cells is required to derive critical cellular components such as amino acids, nucleotides and lipids from glycolytic intermediates. Efficient ATP production by oxidative phosphorylation (OxPhos) is also critical for cancer cells, and compounds which target this key process are being explored as cancer therapeutics [14]. Interestingly, the antidiabetic biguanide drug metformin, which is a mild inhibitor of complex I of the electron transport chain (ETC) [15], and thus OxPhos, has significant anticancer properties [16, 17]. However it is unclear whether these beneficial effects of metformin in cancer patients are tumour cell autonomous, or are rather the systemic effects of mitigating the deleterious symptoms of metabolic syndrome [18]. Nevertheless, clinical trials testing the efficacy of metformin in a wide range of cancers have been undertaken [14]. OxPhos inhibitors with increased potency and cellular uptake, such as the discontinued biguanide drug phenformin, are also being tested in preclinical cancer models [19, 20].

Dominantly-acting oncogenes have been shown to drive increased glycolysis in tumour cells [21, 22]. In this study, we hypothesized that osimertinib could inhibit glycolysis in EGFR-mutant lung adenocarcinoma lines. Moreover, we wished to test whether osimertinib resistance led to an altered metabolic phenotype, and critically, whether OxPhos inhibition could inhibit the development of osimertinib resistance in EGFR mutant lung cancer lines.

## RESULTS

**Figure 1:**
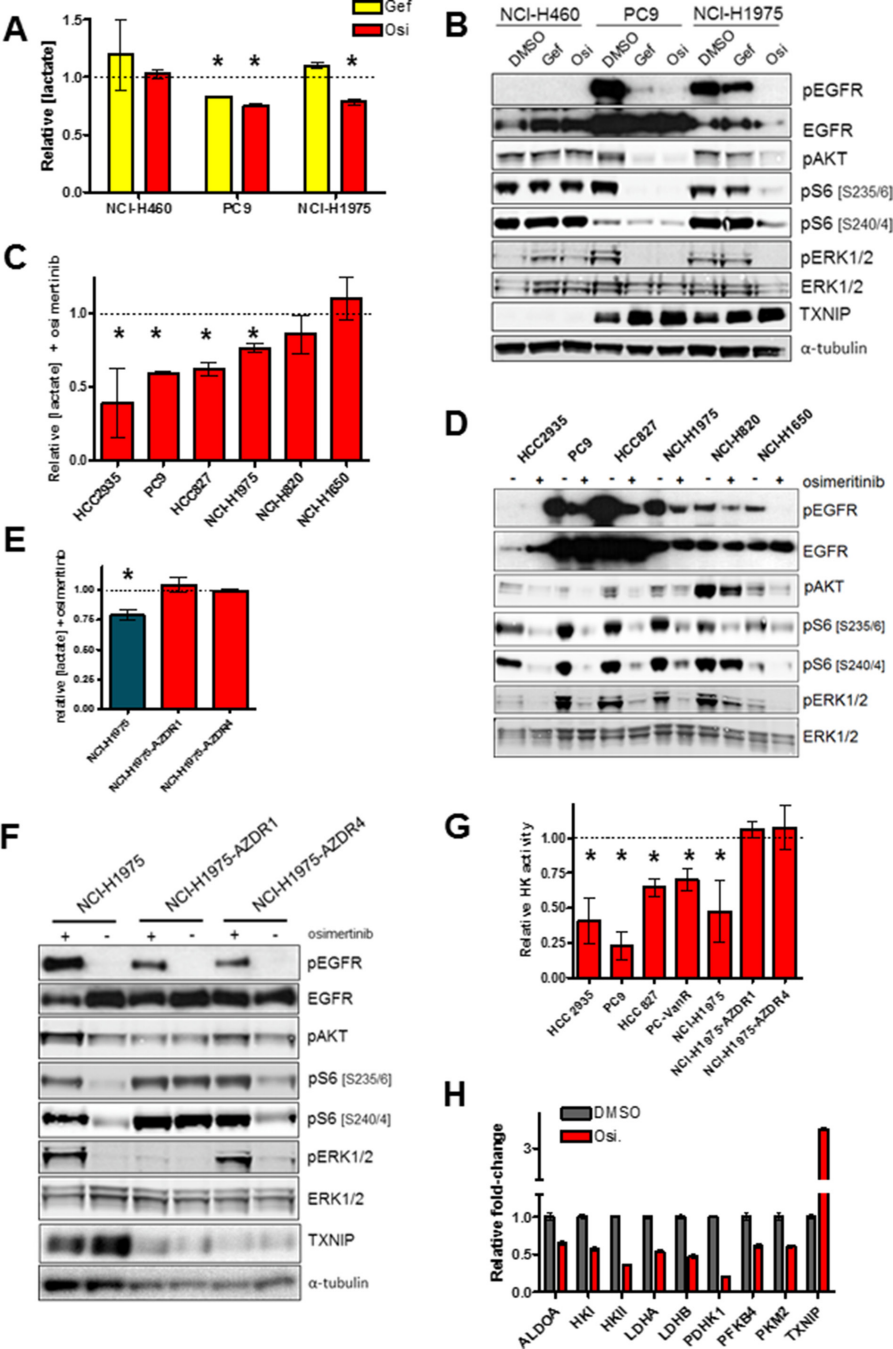
Osimertinib blocks glycolysis in sensitive but not resistant cells (**A**) Cells were plated and treated with either vehicle control, 300 nM gefinitib (Gef) or 160 nM osimertinib (Osi) for 24 h. Conditioned media was analysed for lactate concentration, values shown are means relative to vehicle control +/− SEM (*p < 0.05; *n* = 3). (**B**) Cells treated as in (A), lysed and subjected to Western blotting. (**C**) Cells were treated with 160 nM osimertinib or vehicle control for 24h, and conditioned media was analysed for lactate concentration. Values shown are means relative to vehicle control +/− SEM (*p < 0.05; *n* ≥ 3). (**D**) Cells were treated as in (C), lysed and subjected to Western blotting. (**E**) Cells were treated with 160 nM osimertinib or vehicle control for 24 h, and conditioned media was analysed as in (A) (*n* = 3). (**F**) Cells were treated as in (E), lysed and subjected to Western blotting. (**G**) Cells were treated with 160 nM osimertinib or vehicle control for 24 h, cells were lysed and subjected to an enzymatic hexokinase assay. Results were normalized to total protein level and values shown are means relative to vehicle control (*p < 0.05; *n* = 3). (**H**) PC9 cells were treated with 160 nM osimertinib or vehicle control for 24 h, RNA was isolated and relative levels of mRNA expression was determined by qPCR. Values shown are means relative to vehicle control +/− SEM (*n* = 3). All osimertinib-treated samples showed significant differences from DMSO control (*p* < 0.05).

Previous reports have shown that suppression of dominantly-acting kinase oncogenes via specific small molecule inhibitors can attenuate glycolysis [21, 22]. We wished to evaluate the effects of osimertinib on glycolytic metabolism in EGFRm lung cancer cell lines. As seen in Figure 1A, osimertinib impairs lactate production – a marker of glycolysis - in the EGFR-mutant lines NCI-H1975 and PC9, while having no effect on lactate in the EGFR-wildtype line NCI-H460. This effect is EGFR-specific, because gefitinib inhibits glycolysis in PC9 cells, but not in gefitinib-resistant T790M+ NCI-H1975 cells which retain EGFR activity (Figure 1B). Osimertinib inhibited lactate secretion in an expanded set of EGFRm lines, with the exception of NCI-H1650 cells (Figure 1C), which are intrinsically resistant to EGFR inhibitors including osimertinib (reference [23] and Supplementary Figure S1A), and only modestly suppress S6 phosphorylation in response to osimertinib despite EGFR suppression (Figure 1D). Overall there was a significant inverse correlation between glycolysis inhibition and both average osimertinib IC50 and suppression of S6 phosphorylation (Supplementary Figure S1B, S1C and Supplementary Table S1). Interestingly, NCI-H1975 cell lines with acquired osimertinib resistance [24] cultured in the absence of osimertinib showed no change in glycolysis after subsequent osimertinib treatment (Figure 1E), despite potent EGFR inhibition (Figure 1F). Similar results were obtained in osimertinib-resistant derivatives of PC9 cells, where osimertinib treatment either has no (PC9-AZDR1 and PC9-AZDR2) or little (PC9-AZDR4) effect on lactate secretion (Supplementary Figure S1D). We confirmed that osimertinib's effects on lactate were due to impairment of glycolysis by measuring hexokinase activity – the first enzymatic step in glycolysis - on similarly treated cells. We saw that osimertinib sensitive cells showed reduced hexokinase activity after drug treatment, whereas NCI-H1975 cells with acquired osimertinib resistance did not alter hexokinase activity in response to EGFR inhibition (Figure 1G) Taken together these data suggest that osimertinib-resistant cells rely on an alternative pathway for glycolysis in the absence of EGFR. However cells with T790M-mediated acquired resistance to gefitinib (PC9-IR4) or vandetinib (PC9-VanR) showed attenuated lactate secretion after osimertinib treatment (Supplementary Figure S1E), aligned with their growth sensitivity to this drug (Supplementary Figure S1F). Moreover, PC9-VanR cells showed reduced hexokinase activity after osimertinib treatment (Figure 1G). Gene expression analysis of osimertinib-sensitive PC9 cells showed that several key regulators of glycolysis were downregulated after osimertinib (Figure 1H). Downregulation glycolytic genes was confirmed at the mRNA level in a panel of osimertinib-sensitive lines, particularly evident in HCC827 and PC9-VanR cells, whereas osimertinib-resistant NCI-H1975 cells showed no significant downregulation of this set of mRNAs (Supplementary Figure S1G). Moreover, 24h osimertinib treatment of PC9, PC9-VanR and NCI-H1975 cells caused a significant increase in mRNA expression of thioredoxin interacting protein (TXNIP), a negative regulator of glycolysis [25] (Supplementary Figure S1H and S1G), which was confirmed at the protein level in 3 of 4 osimertinib-sensitive cell lines, but not in resistant cells (Supplementary Figure S1H). Therefore we conclude that inhibition of glycolysis correlates with growth inhibition by osimertinib, and that glycolysis in cells with acquired osimertinib resistance is EGFR-independent.

**Figure 2:**
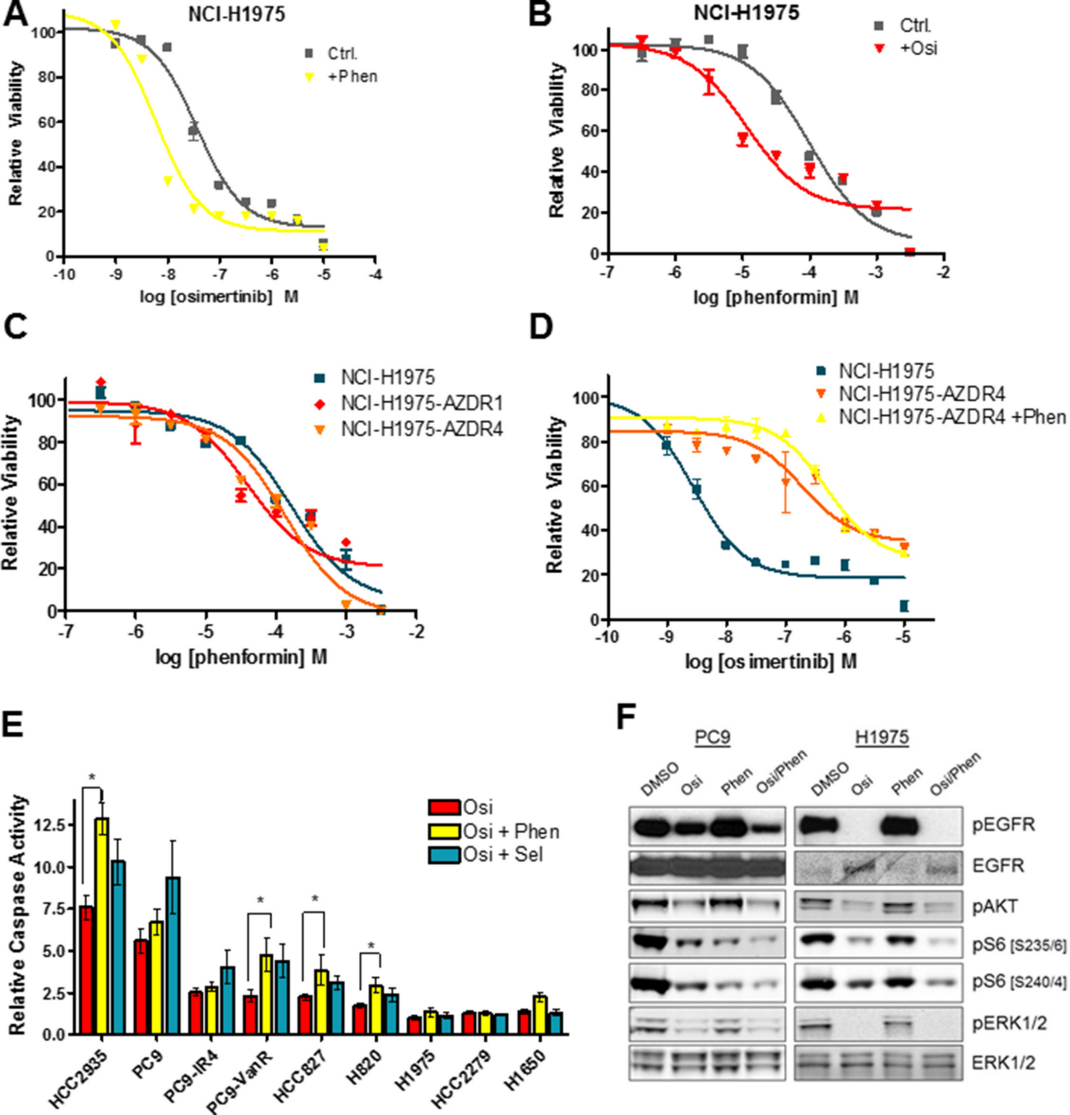
The osimertinib/phenformin combination suppresses signaling to the S6 ribosomal pathway, but does not show synergistic growth inhibition in short-term assays (**A**) Growth response curve of NCI-H1975 cells for osimertinib in the presence or absence of 0.1 mM phenformin (+Phen). (**B**) Growth response curve of NCI-H1975 cells for phenformin in the presence of absence of 160 nM osimertinib (+Osi). (**C**) Growth response curve of NCI-H1975, NCI-H1975-AZDR1 and NCI-H1975-AZDZR4 cells to phenformin. (**D**) Growth response curve to osimertinib of NCI-H1975, NCI-H1975-AZDR4, and NCI-H1975-AZDR4 cells co-treated with 0.1 mM phenformin. (**E**) Cells were treated for 24 h with 160 nM (PC9, PC9-GR6, PC9-VanR, HCC2935, HCC827, NCI-H1975) or 300 nM osimertinib alone or in combination with 300 nM selumetinib or 30 μM (PC9, PC9-GR6, PC9-VanR) or 0.1 mM phenformin and subjected to a caspase 3/7 assay as described in the Materials and Methods. Values shown are means relative to vehicle control +/− SEM (*p < 0.05; *n* ≥ 3). (**F**) Cells were treated for 24 h with either osimertinib (Osi; PC9 - 20 nM; NCI-H1975 – 160 nM), phenformin (PC9 – 30 μM; NCI-H1975 – 0.1 mM) or the combination of both. Cells were lysed and subjected to Western blotting.

Because osimertinib effectively inhibits glycolysis in sensitive lines, we hypothesized that OxPhos inhibitors might synergize with osimertinib to block the growth of EGFR-mutant lines. We compared osimertinib growth response curves with and without the addition of 0.1 mM phenformin (approximate IC50). Phenformin had no effect on the acute osimertinib sensitivity of PC9 and HCC827 cell lines (Supplementary Figure S2A, S2B), whereas phenformin caused a modest increase in the osimertinib sensitivity of NCI-H1975 cells (Figure 2A). Conversely, the sensitivity of NCI-H1975 cells to a range of OxPhos inhibitors including phenformin (Figure 2B), metformin (Figure S2C), the biguanide buformin (Supplementary Figure S2D) and BAY 87-2243 - a small molecule with potent OxPhos inhibitory properties [26] - (Supplementary Figure S2E) was enhanced by the addition of osimertinib. Further evidence co-suppression of glycolysis and OxPhos can impair growth of EGFRm cells was provided by the fact that oligomycin, an ATP synthase inhibitor that blocks OxPhos, sensitizes NCI-H1975 cells to 2-DG, a glucose analogue that blocks the glycolytic pathway (Supplementary Figure S2F). Because osimertinib fails to suppress glycolysis in resistant cells, we hypothesized that they would show no increased sensitivity to OxPhos inhibition compared to sensitive lines, and indeed there is no significant increase in sensitivity to phenformin (Figure 2C, Supplementary S2G), metformin (Supplementary Figure S2H) or BAY-87-2243 (Supplementary Figure S2I). Furthermore, phenformin co-treatment had no effect on osimertinib sensitivity of resistant cells (Figure 2E). When NCI-H1975, PC9 and HCC827 cells were treated with osimertinib or phenformin alone or in combination (Figure 2F, S2J), osimertinib effectively inhibited ERK1/2 phosphorylation, which was not altered by the addition of phenformin. However, ribosomal S6 protein phosphorylation was more strongly inhibited by osimertinib/phenformin treatment than treatment with either drug alone. We next hypothesized that concurrent inhibition of glycolysis and OxPhos metabolism might induce apoptosis in EGFRm cells. The osimertinib/phenformin combination significantly increased caspase 3/7 activation in a subset of cell lines (NCI-H820, HCC827, HCC2935 and PC9-VanR) over osimertinib alone (Figure 2G, Supplementary Figure S2K), and we observed a similar pattern when a subset of these cell lines were subjected to Annexin V staining after compound treatment (Supplementary Figure S2L). We observed a similar proportion of lines that showed increased caspase activity upon addition of the MEK inhibitor selumetinib (AZD6244, ARRY-142886), as previously described [23], though these two sets of cell lines did not completely overlap.

**Figure 3:**
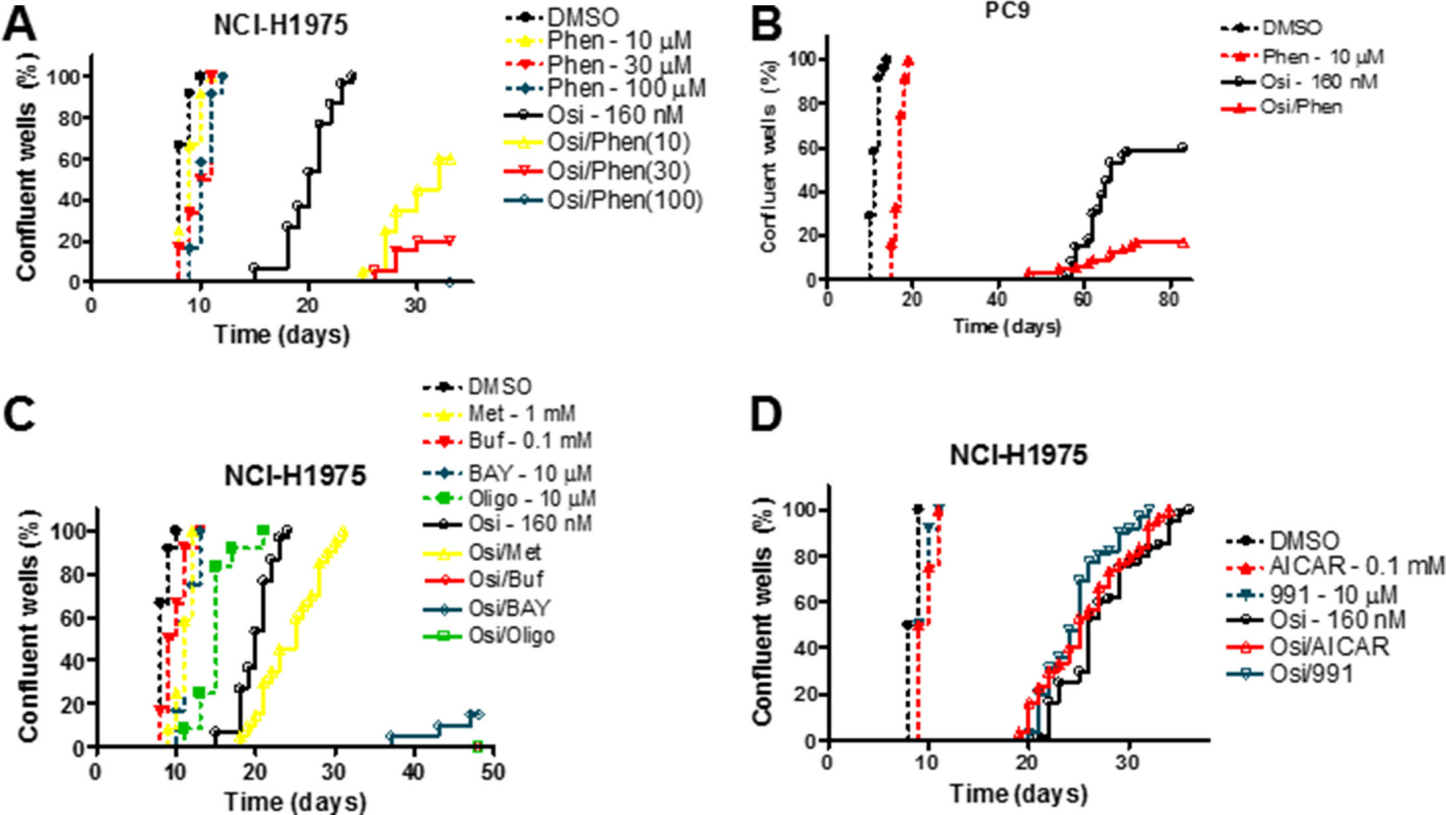
Inhibitors of oxidative phosphorylation inhibit the development of osimertinib resistance in an AMPK-independent manner (**A**–**D**) Cells were plated at low density, treated with the indicated compounds and scored for resistance as described in the Materials and Methods. Abbreviations: osimertinib (Osi), phenformin (Phen), metformin (Met), buformin (Buf), BAY 87-2243 (BAY), oligomycin (Oligo).

Although we saw an increased sensitivity to osimertinib in NCI-H1975 cells with concurrent phenformin treatment in a short-term growth assay, we wished to assess the effect of OxPhos inhibitors on the acquisition of osimertinib resistance in a model that better represents clinical progression. We employed a long-term culture system where cells were plated at low density in multiple wells and cultured for a prolonged period [23]. In this assay, all osimertinib-treated NCI-H1975 wells achieved resistance by day 25 post-plating (Figure 3A), whereas more sensitive cell lines had both a lower proportion of resistant wells and a longer median time to resistance (Figure 3A, Supplementary Figure S3A–S3C). Nevertheless, for all cell lines tested, phenformin delayed or prevented the development of osimertinib resistance in a dose dependent manner. For example, in PC9 cells only 10% of wells (3/30) became resistant when 30 nM osimertinib was combined with 30 μM phenformin compared to 97% of wells (29/30) with osimertinib monotherapy (Supplementary Figure S3A). At 160 nM osimertinib (Figure 3B), 60% of wells (18/30) reached confluence, which was reduced to 17% (5/30) with the addition of phenformin. Similarly, in NCI-H1975 cells 100 μM phenformin, a dose that had negligible effects as monotherapy, blocked resistance to both osimertinib (Figure 3A) and CO-1686, another T790M-targeting EGFR inhibitor [27]; Supplementary Figure S3D). In contrast, in NCI-H1650 cells, which show EGFR-independent glycolysis (Figure 1C), phenformin had only modest effects when combined with osimertinib (Supplementary Figure S3E), supporting the hypothesis that simultaneous inhibition of glycolysis and OxPhos mediates attenuation of resistance. Further, combining osimertinib and a panel of OxPhos inhibitors (metformin, buformin, BAY 87-2243 and oligomycin) in NCI-H1975 cells showed a dramatic suppression or elimination of resistance, aside from metformin which had only modest effects commensurate with its previously described lower potency in transformed cells [28] (Figure 3C). Finally, we established that the effects of OxPhos inhibition on resistance were AMPK-independent, as the direct AMPK activators compound 991 [29] and 5-Aminoimidazole-4-carboxamide ribonucleotide (AICAR) were unable to alter the kinetics of osimertinib resistance (Figure 3D).

**Figure 4:**
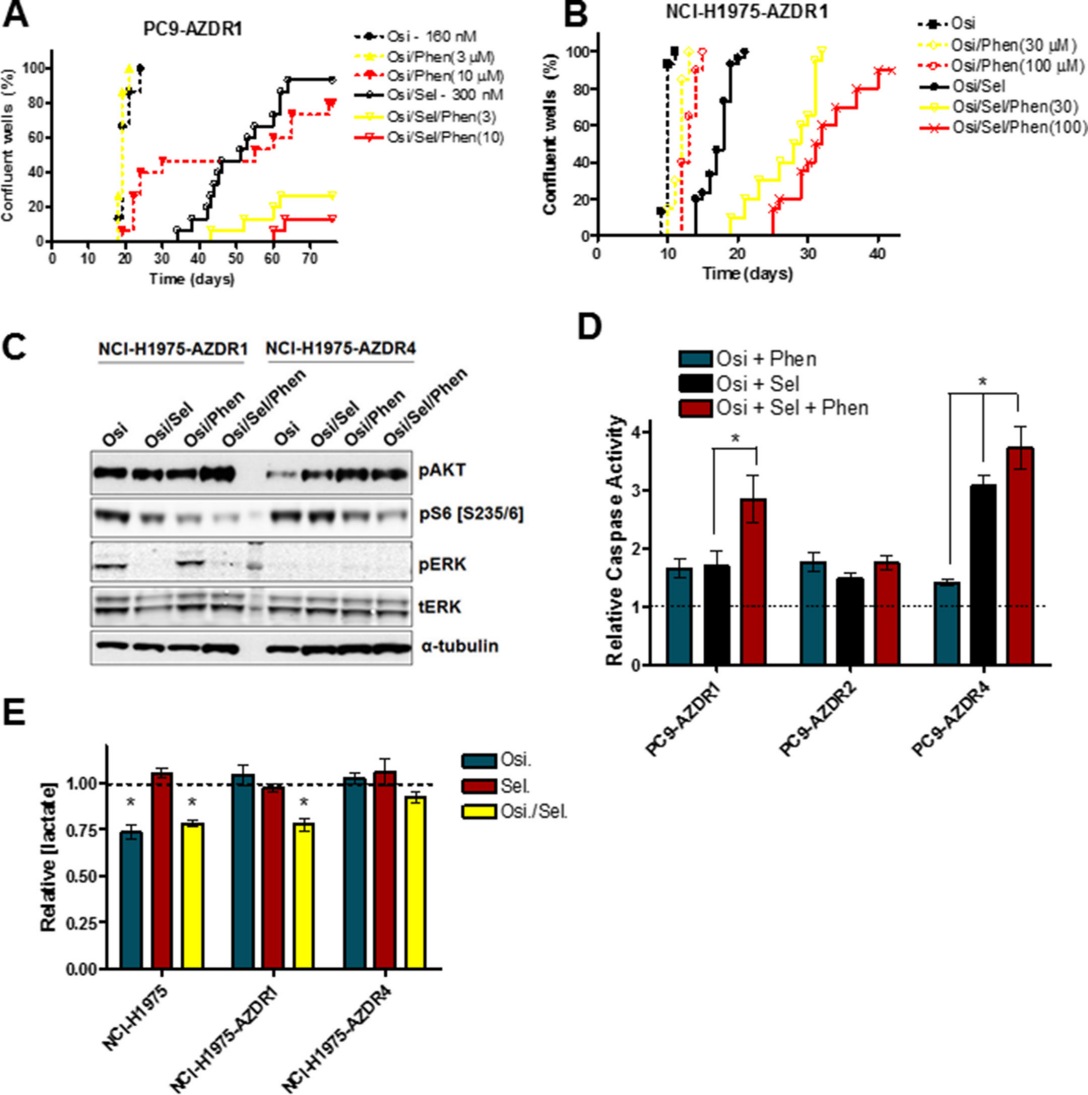
Phenformin inhibits the development of selumetinib resistance in osimertinib resistant cell lines PC9-AZDR1 (**A**) and NCI-H1975-AZDR1 (**B**) cells were plated at low density, treated with the indicated compounds and scored for resistance as described in the Materials and Methods. (**C**) NCI-H1975-AZDR1 and NCI-H1975-AZDR4 cells were treated as follows, lysed and subjected to Western blotting; lane 1. 160 nM osimertinib; lane 2. Osimertinib/300 nM selumetinib; lane 3. Osimertinib/100 μM phenformin; lane 4. Osimertinib/ selumetinib/phenformin. (**D**) Indicated cell lines were treated for 48 h with osimertinib (160 nM) + selumetinib (300 nM), osimertinib + phenformin (30 μM) or osimertinib + selumetinib + phenformin and subjected to a caspase 3/7 assay as described in the Materials and Methods. Values shown are means relative to osimertinib-only control +/− SEM (*p < 0.05; *n* = 3). (**E**) Cells were treated for 24 h with osimertinib (Osi; 160 nM), selumetinib (Sel; 300 nM) or both (Osi/Sel), and conditioned media was analysed for lactate concentration. Values shown are means relative to osimertinib-only control +/− SEM (*p < 0.05; *n* = 3).

Previous reports have shown that a significant subset of osimertinib-resistant cell lines show markedly enhanced sensitivity to MEK inhibition [23, 24]. However we predict that resistance will develop after initial MEK inhibitor sensitivity. To model this phenomenon *in vitro*, selumetinib-sensitive PC9-AZDR1 and NCI-H1975-AZDR1 cells were plated in long-term culture assays as described above (Figure 4A, 4B). After initial sensitivity to MEK inhibition by selumetinib, 93% and 100% of PC9-AZDR1 wells demonstrated resistance (Osi/Sel treatment). In contrast, co-treatment of PC9-AZDR1 cells with osimertinib, selumetinib and 3 or 10 μM phenformin (Osi/Sel/Phen treatment) resulted in only 27% (8/30) or 13% (4/30) of resistant wells, respectively. Similar results were seen for a separate osimertinib-resistant, MEKi-sensitive line (PC9-AZDR4 cells; Supplementary Figure S4A). Moreover, while phenformin did not alter the proportion of wells demonstrating resistance to Osi/Sel treatment in NCI-H1975-AZDR1 cells, it significantly delayed the median onset of time to resistance from 18 days to 28.5 days (30 μM phenformin) or 31.5 days (100 μM phenformin). Importantly, PC9-AZDR2 and NCI-H1975-AZDR4 cells, which show no enhanced MEK inhibitor sensitivity in short-term assays [24] showed only moderate sensitivity to the Osi/Sel combination and the addition of phenformin has minimal effects on resistance (Supplementary Figure S4B, S4C). PC9-AZDR1, PC9-AZDR4, NCI-H1975-AZDR1 and NCI-H1975-AZDR4 cells treated with the Osi/Sel/Phen combination showed an enhanced inhibition of S6 phosphorylation compared to Osi/Sel or Osi/Phen treatment (Figure 4C, Supplementary Figure S4C). Furthermore, the addition of phenformin potentiated the apoptotic effect of Osi/Sel in PC9-AZDR1 cells, while the effects in PC9-AZDR2 cells were minimal (Figure 4D). NCI-H1975-AZDR1 cells showed minimal apoptosis after Osi/Sel treatment similar to the parental cell line (Figure 2E) that could not be enhanced by phenformin (data not shown). Our model predicts that the ability of phenformin to suppress Osi/Sel resistance in PC9-AZDR1, PC9-AZDR4 and NCI-H1975-AZDR1 cells is due to its OxPhos inhibitory effects. We see that while osimertinib alone had no effect on lactate production in PC9-AZDR1, PC9-AZDR4 and NCI-H1975-AZDR1 cells, Osi/Sel effectively inhibits glycolysis in these cells (Figures 4E, S4D), whereas this combined inhibition of EGFR and MEK has a limited effect on insensitive PC9-AZDR2 and NCI-H1975-AZDR4 cells. Taken together these data support the idea that simultaneous suppression of glycolysis and OxPhos underlies the cooperative effects of phenformin and small molecule kinase inhibitors.

**Figure 5:**
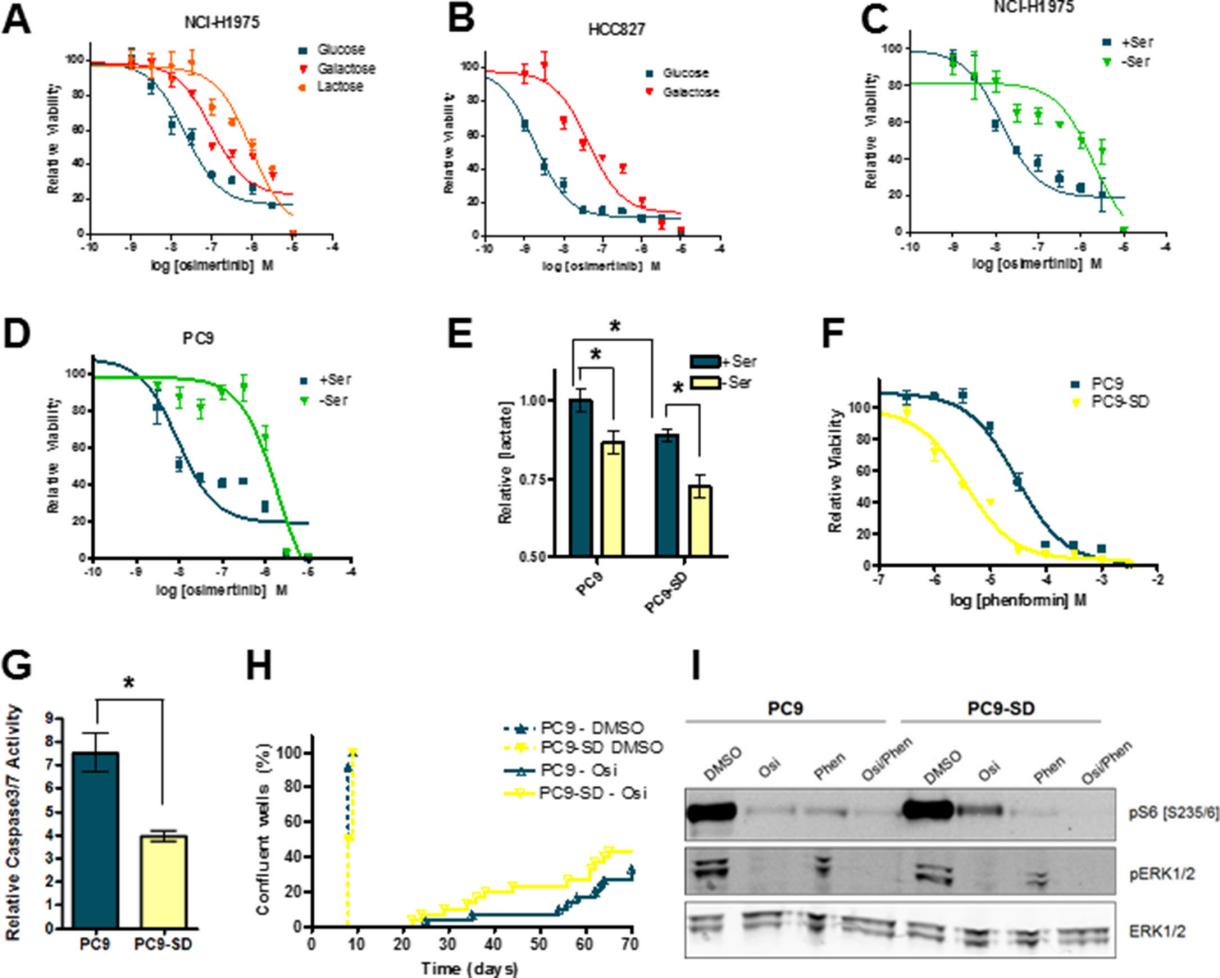
Growth conditions which promote OxPhos confer a degree of osimertinib resistance to EGFRm cell lines (**A**) Growth response curve for NCI-H1975 cells grown in DMEM containing 5 mM glucose, galactose or lactose. (**B**) Growth response curve for HCC827 cells grown in DMEM containing 5 mM glucose or galactose. Osimertinib growth response curve for NCI-H1975 (**C**) or PC9 (**D**) cells grown in either SFM (-Ser) or SFM with serine and glycine added back (+Ser). (**E**) PC9 and PC9-SD cells were grown in either -Ser or +Ser for 24h, and conditioned media was analysed for lactate concentration. Values shown are means relative to PC9 cells grown +serine +/− SEM (*p < 0.05; *n* = 3). (**F**) Osimertinib growth response curve for PC9 vs. PC9-SD cells grown in RPMI medium. (**G**) PC9 and PC9-SD cells were treated with 160 nM osimertinib or vehicle control for 48 h in RPMI medium and subjected to a caspase 3/7 assay as described in the Materials and Methods. Values shown are means relative to vehicle control +/− SEM (*p < 0.05; *n* = 3). (**H**) PC9 and PC9-SD cells were plated at low density, treated with the 160 nM osimertinib or vehicle control and scored for resistance as described in the Materials and Methods. (**I**) PC9 and PC9-SD cells were treated with vehicle control, 160 nM osimertinib, 30 μM phenformin or an osimertinib/phenformin combination for 24 h. Cells were lysed and subjected to Western blotting.

We hypothesize here that inhibition of OxPhos suppresses the development of osimertinib resistance in EGFR-mutant lung cancer cell lines. We next tested whether cells with enhanced OxPhos activity showed increased osimertinib resistance. OxPhos can be upregulated by altering the main fuel source in cells from glucose to lactose or galactose, which forces cells to generate ATP via mitochondrial respiration. As expected, NCI-H1975 cells plated in galactose showed enhanced sensitivity to phenformin (Supplementary Figure S5A), but interestingly were significantly less sensitive to osimertinib (Figure 5A). Similarly, HCC827 cells showed marked osimertinib resistance when grown in galactose media (Figure 5B), while PC9 cells showed reduced osimertinib sensitivity in lactate-containing media (Supplementary Figure S5B). OxPhos can also be enhanced by serine deprivation, as cells divert the glycolytic intermediate 3-phosphoglycerate into the *de novo* serine synthesis pathway. In serine/glycine-depleted conditions (SFM), 3 of 4 cell lines showed marked osimertinib resistance (Figure 5C, 5D and Supplementary S5C, S5D). To explore this phenomenon further, we generated “serine-deprived” (SD) derivatives of PC9 cells by prolonged culture (> 21 days) in SFM (Supplementary Figure S5E). These cells showed reduced glycolysis which persisted when serine was added back to culture (Figure 5E). Moreover, they showed enhanced sensitivity to phenformin when grown in full RPMI media (Figure 5F). While no enhanced osimertinib resistance was observed in PC9-SD cells in short-term growth assays (Supplementary Figure S5F), they displayed significantly less osimertinib-induced apoptosis (Figure 5G). Critically, when these cells were plated in long-term resistance assays, PC9-SD cells showed significantly earlier onset of resistance (Figure 5H). Interestingly, PC9-SD cells maintain a higher degree of S6 phosphorylation after osimertinib treatment compared to parental cells (Figure 5I). Thus when EGFR-mutant cells derive their ATP via OxPhos rather than glycolysis they show decreased osimertinib sensitivity, and increased sensitivity to OxPhos inhibitors.

**Figure 6:**
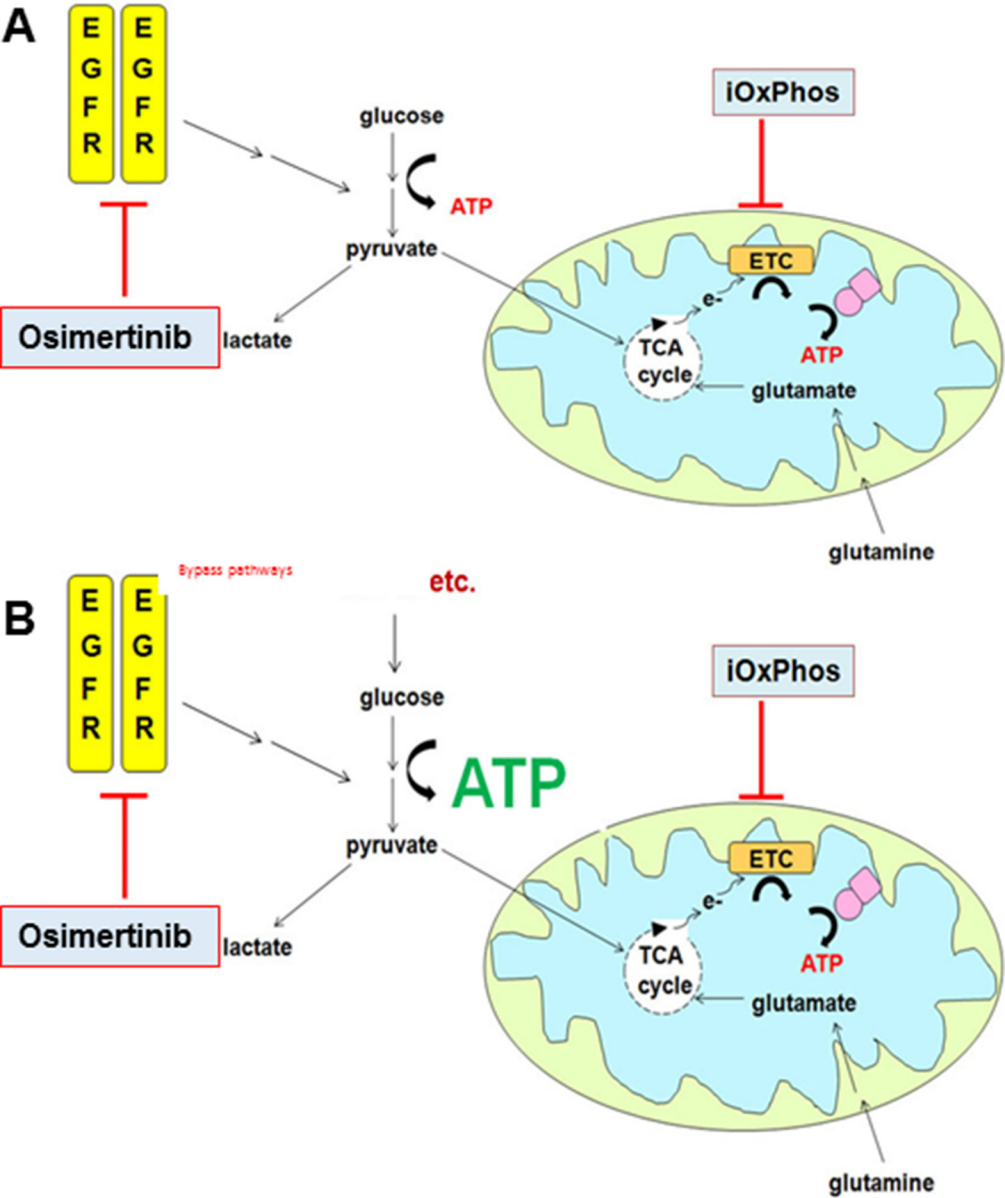
Combined inhibition of glycolysis and OxPhos suppresses osimertinib resistance (**A**) In osimertinib-sensitive cells, glycolysis is EGFR-dependent. Concurrent inhibition of OxPhos induces metabolic crisis and cells are significantly less able to develop osimertinib resistance. (**B**) In osimertinib-resistant cells, glycolysis is driven by EGFR-independent bypass pathways. In combination treatment, OxPhos is inhibited yet cells can produce ATP via glycolysis. Overall OxPhos inhibitor sensitivity is similar to that of osimertinib-sensitive cells treated with OxPhos inhibitor monotherapy.

## DISCUSSION

Osimertinib provides an effective treatment option for NSCLC patients with EGFRm-positive tumours that have relapsed while on EGFR TKI therapy due to T790M. Moreover, osimertinib has been recently tested in the first-line setting for EGFRm patients – a majority of which were T790M-negative – and was found to have a promising median progression-free survival of 19.3 months [30]. However, osimertinib and other TKI monotherapies are not curative, so additional therapeutic strategies must be identified to prolong patient benefit. Potential diverse clinical molecular mechanisms of EGFR TKI resistance are increasingly becoming identified. For instance, T790M [1], MET [31] or HER2 [32] amplification and PIK3CA mutation [5] have been associated with resistance to early generation TKIs. Similarly, clinical resistance mechanisms to osimertinib include EGFR-C797S mutation [10] or bypass signalling through MET or HER2 [33]. This provides a challenge of identifying molecular resistance segments that would respond to novel targeted combinations. However, from the data presented here, we hypothesize that by subjecting tumour cells to apoptotic or metabolic stress through dual blockade of glycolysis and OxPhos, the pool of actively dividing cells with the potential to develop such resistance mechanisms would be significantly reduced. Moreover, since our data demonstrates that such a strategy can be effective across different cell backgrounds and resistance mechanisms, this approach could provide a more generic strategy to combat resistance in heterogeneous tumours. Interestingly, after we generated osimertinib-resistant cell lines *in vitro*, we found them to be equally sensitive to OxPhos inhibitors as their parental counterparts, even when osimertinib is present. This finding is critical for devising a potential therapeutic strategy, suggesting that maximal patient benefit would occur with the osimertinib/OxPhos inhibitor combination given up front in osimertinib-sensitive tumours, whereas such a combination would provide little benefit once resistance was established in a tumour population.

We [24] and others [23] have previously shown that combining EGFR and MEK inhibitors can significantly impair resistance to EGFR inhibition in EGFRm positive NSCLC cell lines Moreover, we have shown that a subset of cell lines with acquired osimertinib display enhanced sensitivity to the MEK inhibitor selumetinib [24]. However resistant cell lines that initially show sensitivity to the osimertinib/selumetinib combination show rapid regrowth both *in vitro* (Figure 4A, 4B) and *in vivo* (data not shown). While mechanisms mediating this “double resistance” are unknown, its rapid onset likely precludes genetic alterations, whereas metabolic adaptation is one possible explanation for the observed results. Phenformin's ability to inhibit osimertinib/selumetinib resistance lends support to this hypothesis, although more careful metabolic analysis of “double resistant” lines is warranted.

Recent interest in the anticancer potential of metformin has exploded, however results have been largely inconclusive with regard to clinical benefit [14]. Metformin requires active transport into cells via the organic cation transporter (OCT1) [34], the expression of which is typically low or absent in transformed cells [35]. In our current study, we saw that of OxPhos inhibitors tested, metformin was the least proficient at inhibiting osimertinib resistance (Figure 3C), even at supraclinical doses. Although discontinued in the diabetes setting, phenformin has advantages over metformin as an oncology drug; it does not require OCT1-mediated transport into cells [35] and is a more potent inhibitor of complex I [15]. Phenformin's primary adverse effect is severe lactic acidosis in a subset of patients [36], but there is evidence that susceptibility is genetically determined [37], and early screening to identify vulnerable patients, combined with careful monitoring of blood lactate could mitigate the potential for adverse effects. Thus this drug holds promise for cancer therapy, particularly in the settings of LKB1-null lung cancer [20] and combination strategies such as those proposed by our study and others [38]. However data presented here and elsewhere [39] also points to the therapeutic potential of small-molecule complex I inhibitors such as BAY 87-2243 that have potency in the high nanomolar range, supporting further drug development efforts for this class of complex I inhibitors that may have a more favourable safety profile.

The results of this study are potentially relevant not only to EGFR-driven lung adenocarcinoma, but also to other cancers where glycolysis is driven by dominantly-acting oncogenes. In particular, these data resemble the metabolic adaptations in BRAF-mutant melanoma cells treated with pathway-specific inhibitors where lactate production is suppressed [21, 40] and mitochondrial protein levels increased [40]. We note that a recent report showed dramatic upregulation of mitochondrial ETC proteins after treatment of EGFR-mutant cells with pathway inhibitors [41]. However, we failed to detect any osimertinib-mediated upregulation in mitochondrial ETC genes at the mRNA (data not shown), or protein (Supplementary Figure S1E) level. We did observe a modest increase in basal oxygen consumption in PC9 cells pre-treated with osimertinib (Supplementary Figure S1J), although similar treatment in NCI-H1975 cells caused no alteration of basal oxygen consumption (Figure S1K). Notably, the addition of FCCP showed that the maximal respiration rate is roughly equivalent to the basal respiration rate, even in vehicle control cells. Similarly, osimertinib-resistant lines maintained basal respiration at near-maximal levels, which was not significantly altered by either osimertinib or selumetinib treatment (Supplementary Figure S1L, S1M and Supplementary Figure S4F, S4G). Based on our data, we postulate that OxPhos activity is maintained at a high level in untreated EGFRm cells, and after osimertinib-induced glycolysis suppression cells become strongly dependent on OxPhos metabolism for energy generation. Thus the osimertinib/phenformin combination abrogates the metabolic “escape route” provided by OxPhos activity and cells are effectively prevented from developing osimertinib resistance.

Our compelling *in vitro* data showing inhibition of osimertinib resistance by concurrent treatment with OxPhos inhibitors prompts the question as to whether this can be replicated *in vivo*. Unfortunately modelling osimertinib resistance *in vivo* is difficult due to the potency of osimertinib against EGFR-mutant xenografts, where under continuous dosing no resistance is observed [8]. To circumvent this issue, we treated animals bearing NCI-H1975 xenografts with osimertinib alone or in combination with phenformin, metformin or selumetinib for 28 days, and then monitored animals for tumour regrowth (Supplementary Figure S6). Animals treated with osimertinib alone exhibited regrowth beginning approximately 50 days after treatment initiation. Combining osimertinib with phenformin led to a decreased average tumour size at day 63 over osimertinib alone that was on the verge of statistical significance (*p* = 0.054; one-way ANOVA). Similar results were obtained with metformin treatment. Notably, selumetinib in combination with osimertinib did not improve upon the osimertinib/phenformin combination in this model despite the osimertinib/selumetinib combination being very effective in a transgenic model of EGFR-driven lung cancer [24]. We conclude that this regrowth model, which is dependent on drug cessation, is not optimal for modelling resistance in patients, and future work testing the osimertinib/phenformin combination in established transgenic models will be very informative.

In summary, we have shown that the EGFR T790M inhibitor osimertinib suppresses glycolysis, and simultaneous treatment with OxPhos inhibitors delays or prevents the emergence of drug resistance. Further pre-clinical studies, for example in genetically-defined mouse models of EGFR T790M-driven lung adenocarcinoma, will be key for translating this preclinical finding to a potential therapeutic opportunity. We propose that this could be a broadly applicable combination strategy that enhances the benefit of osimertinib and other EGFR TKIs in EGFRm NSCLC patients.

## MATERIALS AND METHODS

### Cell culture

All cell lines are listed in Supplementary Table S2. PC9-SD cells were derived from PC9 parental cells by growth in serine/glycine-free media (SFM) for 21 days. The identity of all cell lines was confirmed by short tandem repeat (STR) profiling. All cells were grown in RPMI1640 (Gibco), supplemented with 10% fetal calf serum (FCS) and 2 mM glutamine, with the exception of the experiments where media manipulations were carried out as follows: SFM – minimal essential medium (MEM; Gibco Cat. ♯ 21090), 1× MEM vitamins (Gibco Cat. ♯ 11120), 10% dialysed FCS (Hyclone) and 2 mM glutamine; for controls serine and glycine were added back to the above media at a concentration of 0.4 mM; galactose/lactose-containing media – DMEM no glucose, no glutamine (Gibco Cat. ♯ A14430-01), supplemented with 10% dialysed FCS, 2 mM glutamine and either 5 mM glucose (control), galactose or lactose.

### Reagents and western blotting

Osimertinib, selumetinib and gefitinib were synthesized according to published methods. Phenformin, metformin, 2-deoxyglucose and oligomycin were purchased from Sigma. Buformin was purchased from Santa Cruz. BAY-87-2243 was purchased from Selleck Chemicals. All antibodies used are listed in Supplementary Table S3. Culture medium was aspirated from cells and cells were washed once in cold PBS. Cells were scraped into 100μl lysis buffer (25 mM Tris-HCl (pH 6.8), 3 mM EDTA, 5 mM EGTA, 0.27 M sucrose 0.5% Triton X-100, 50 mM NaF, 2 mM Na3VO4, 10 mM beta-glycerophosphate, 5 mM sodium pyrophosphate, Complete protease inhibitor tablets (Roche)) per 35 mm dish. Protein concentrations were determined by the bicinchoninic acid (BCA) protocol from Pierce and Western blots were performed by running samples of equal protein concentration on SDS-PAGE gels (NuPAGE^™^ Novex^™^ 4–12% Bis-Tris Protein Gel, Thermo Scientific), transferring proteins to polyvinylidene fluoride membranes, incubating with primary antibodies overnight, followed by addition of A) fluorescent-labelled secondary antibodies (Li-COR Biosciences) and analysis on an Odyssey Infrared Scanner (Li-COR Biosciences) or B) HRP-conjugated secondary antibodies (Cell Signaling) and detected with Supersignal Pico West chemiluminescent substrate (Thermo Scientific).

### Real-time PCR

Total RNA was purified from cell lines on a Qiacube using the Qiagen RNeasy Kit. Targeted gene expression was performed using a 48 × 48 Fluidigm dynamic array and ABI primers (Thermo Scientific). 50 ng of total RNA from cell lines was reverse transcribed using a high capacity cDNA reverse transcription kit (Thermo Scientific) and pre-amplified with a Taqman PreAmp master mix (Thermo Scientific) for 14 cycles with 48 selected primers. The Fluidigm Array was then primed and loaded on an IFC Controller and qPCR experiments run on the Biomark System. Data were collected and analysed using the Fluidigm Real-Time PCR Analysis software, generating Ct value. Alternatively, mRNA levels were measured using the QuantiTect Probe RT-PCR kit from Qiagen in 384 well plates run on the LightCycler480 (Roche). Ct values were normalised to an average of TFRC and GAPDH housekeeping genes and treatments were normalized to untreated DMSO controls to calculate Fold Change in gene expression. JMP software was used to calculate *p* values. (*p < 0.05 two-sided paired *t*-test).

### Lactate assay

Cell lines were plated in 12-well dishes at 50,000 cells well. The following day cells were treated in duplicate with compounds in DMEM containing 3 mM glucose, 2 mM glutamine and 10% dialysed FCS (Thermo Scientific). After 24 h, conditioned media was collected, cleared of cellular debris by centrifugation and either frozen at −80°C or immediately subjected to an enzymatic lactate assay (Sigma). Lactate concentrations were calculated compared to a standard curve and values were normalized to overall protein content of each well (BCA assay). Values for each treatment were averaged and normalized to DMSO control, and the experiment was repeated for a minimum *n* = 3.

### Hexokinase assay

Cell lines were plated in 12-well dishes at 50,000 cells/well. The following day cells were treated for 24 h with indicated doses of compound at which time cells were lysed in lysis buffer described above. Lysates were subjected to an enzymatic hexokinase assay (Sigma) according to manufacturer's instructions, normalized to protein content of each well (BCA assay). Values for each treatment were averaged and plotted relative to DMSO control (*n* = 3).

### Oxygen consumption analysis

PC9, PC9-AZDR1, NCI-H1975 and NCI-H1975-AZDR1 cells were plated on custom Seahorse 96-well plates at 20,000 cells/well. The following day cells were treated with osimertinib at the indicated doses for 24 h, at which time cells were washed 2× with unbuffered Seahorse media (supplemented with 10 mM glucose and 1 mM glutamine), and finally incubated with unbuffered media containing compound for 1h at 37°C with 0% CO_<sub>2</sub>_. Plates were then analysed on a Seahorse XF96e bioanalyzer using the Cell Mito Stress Test protocol according to manufacturer's instructions, using 1 μM oligomycin, 1 μM FCCP and 0.5 μM rotenone/antimycin A. Values were normalized to protein concentration as measured by BCA assay after media was removed and cells lysed directly on the plate.

### Cell viability assays

Cells were plated in 96-well plates at 2000 cells/well in 100 μl of media. 24 h later cells were treated in triplicate with a 9-point half-log dosing as well as a DMSO control at 2-fold the final concentration diluted in 100 μl of media. 96 h after dosing cells were analysed using Cell Titer-Glo reagent (Promega) as per the manufacturer's instructions. Values were normalized to DMSO control and plotted using GraphPad Prism. Experiments were repeated for a minimum *n* = 3, and presented graphs represent a typical growth curve.

### Long-term culture

Cells were plated in 96-well plates at 350 cells/well in 100 μl of media. 24 h later cells were treated with compound in either 12 (single compound controls) or 30 wells (osimertinib alone or osimertinib + compound) as well as a DMSO control at 2-fold the final concentration diluted in 100 μl of media. Media was removed and replaced with fresh media + compound (1× dose) every 7 days. Wells were followed daily over time and scored as resistant when they had reached > 80% confluency. The experiment continued until all wells were either confluent or showed no evidence of growth.

### Apoptosis assays

Cells were plated in 96-well plates at 4000 cells/well in 100 μl of media. 24 h later cells were treated with compound in triplicate at 2-fold the final concentration diluted in 100 μl of media. 48 h after dosing plates were analysed. For caspase assays, Caspase 3/7-Glo reagent (Promega) as per the manufacturer's instructions. Duplicate plates were analysed for cell viability by Cell Titer-Glo (see above) simultaneously, and caspase values were normalized to average viability for each treatment. Graphs represent values relative to DMSO for a minimum *n* = 3. For Annexin V staining, cells were double stained with Annexin V fluorescein conjugate/Hoescht for 15 minutes, then read using a 2-channel CellInsight NXT plate reader (ThermoScientific). Results were calculated as % responding cells (Annexin V positive) compared to total number of cell nuclei (Hoescht positive).

### Statistical analyses

Data were expressed as mean ± standard error. Differences were tested by two-tailed *t*-test. The values *P* < 0.05 were considered statistically significant. Statistical analysis was done using GraphPad Prism software (*t*-test). For the *in vivo* experiment, data was analysed using an ANOVA tool developed in-house.

## SUPPLEMENTARY MATERIALS FIGURES AND TABLES



## References

[1] Gazdar AF (2009). Activating and resistance mutations of EGFR in non-small-cell lung cancer: role in clinical response to EGFR tyrosine kinase inhibitors. Oncogene.

[2] Mok TS, Wu YL, Thongprasert S, Yang CH, Chu DT, Saijo N, Sunpaweravong P, Han B, Margono B, Ichinose Y, Nishiwaki Y, Ohe Y, Yang JJ (2009). Gefitinib or carboplatin-paclitaxel in pulmonary adenocarcinoma. N Engl J Med.

[3] Rosell R, Carcereny E, Gervais R, Vergnenegre A, Massuti B, Felip E, Palmero R, Garcia-Gomez R, Pallares C, Sanchez JM, Porta R, Cobo M, Garrido P (2012). Erlotinib versus standard chemotherapy as first-line treatment for European patients with advanced EGFR mutation-positive non-small-cell lung cancer (EURTAC): a multicentre, open-label, randomised phase 3 trial. Lancet Oncol.

[4] Pao W, Miller VA, Politi KA, Riely GJ, Somwar R, Zakowski MF, Kris MG, Varmus H (2005). Acquired resistance of lung adenocarcinomas to gefitinib or erlotinib is associated with a second mutation in the EGFR kinase domain. PLoS Med.

[5] Sequist LV, Waltman BA, Dias-Santagata D, Digumarthy S, Turke AB, Fidias P, Bergethon K, Shaw AT, Gettinger S, Cosper AK, Akhavanfard S, Heist RS, Temel J (2011). Genotypic and histological evolution of lung cancers acquiring resistance to EGFR inhibitors. Sci Transl Med.

[6] Yu HA, Arcila ME, Rekhtman N, Sima CS, Zakowski MF, Pao W, Kris MG, Miller VA, Ladanyi M, Riely GJ (2013). Analysis of tumor specimens at the time of acquired resistance to EGFR-TKI therapy in 155 patients with EGFR-mutant lung cancers. Clin Cancer Res.

[7] Finlay MR, Anderton M, Ashton S, Ballard P, Bethel PA, Box MR, Bradbury RH, Brown SJ, Butterworth S, Campbell A, Chorley C, Colclough N, Cross DA (2014). Discovery of a potent and selective EGFR inhibitor (AZD9291) of both sensitizing and T790M resistance mutations that spares the wild type form of the receptor. J Med Chem.

[8] Cross DA, Ashton SE, Ghiorghiu S, Eberlein C, Nebhan CA, Spitzler PJ, Orme JP, Finlay MR, Ward RA, Mellor MJ, Hughes G, Rahi A, Jacobs VN (2014). AZD9291, an irreversible EGFR TKI, overcomes T790M-mediated resistance to EGFR inhibitors in lung cancer. Cancer Discov.

[9] Goss GD, Yang JCH, Ahn MJ, Tsai CM, Bazhenova L, Sequist LV, Ramalingam SS, Shepherd FA, Ghiorghiu S, Cantarini M, Mann H, Mitsudomi T, Jänne PA (2015). AZD9291 in pre-treated patients with T790M positive advanced non-small cell lung cancer (NSCLC): Pooled analysis from two Phase II studies. The European Cancer Congress.

[10] Thress KS, Paweletz CP, Felip E, Cho BC, Stetson D, Dougherty B, Lai Z, Markovets A, Vivancos A, Kuang Y, Ercan D, Matthews SE, Cantarini M (2015). Acquired EGFR C797S mutation mediates resistance to AZD9291 in non-small cell lung cancer harboring EGFR T790M. Nat Med.

[11] Kim TM, Song A, Kim DW, Kim S, Ahn YO, Keam B, Jeon YK, Lee SH, Chung DH, Heo DS (2015). Mechanisms of Acquired Resistance to AZD9291: A Mutation-Selective, Irreversible EGFR Inhibitor. J Thorac Oncol.

[12] Hanahan D, Weinberg RA (2011). Hallmarks of cancer: the next generation. Cell.

[13] Vander Heiden MG, Cantley LC, Thompson CB (2009). Understanding the Warburg effect: the metabolic requirements of cell proliferation. Science.

[14] Pollak M (2013). Potential applications for biguanides in oncology. J Clin Invest.

[15] Owen MR, Doran E, Halestrap AP (2000). Evidence that metformin exerts its anti-diabetic effects through inhibition of complex 1 of the mitochondrial respiratory chain. Biochem J.

[16] Evans JM, Donnelly LA, Emslie-Smith AM, Alessi DR, Morris AD (2005). Metformin and reduced risk of cancer in diabetic patients. BMJ.

[17] Zhang P, Li H, Tan X, Chen L, Wang S (2013). Association of metformin use with cancer incidence and mortality: a meta-analysis. Cancer Epidemiol.

[18] Martin M, Marais R (2012). Metformin: a diabetes drug for cancer, or a cancer drug for diabetics?. J Clin Oncol.

[19] Huang X, Wullschleger S, Shpiro N, McGuire VA, Sakamoto K, Woods YL, McBurnie W, Fleming S, Alessi DR (2008). Important role of the LKB1-AMPK pathway in suppressing tumorigenesis in PTEN-deficient mice. Biochem J.

[20] Shackelford DB, Abt E, Gerken L, Vasquez DS, Seki A, Leblanc M, Wei L, Fishbein MC, Czernin J, Mischel PS, Shaw RJ (2013). LKB1 inactivation dictates therapeutic response of non-small cell lung cancer to the metabolism drug phenformin. Cancer Cell.

[21] Falck Miniotis M, Arunan V, Eykyn TR, Marais R, Workman P, Leach MO, Beloueche-Babari M (2013). MEK1/2 inhibition decreases lactate in BRAF-driven human cancer cells. Cancer Res.

[22] Makinoshima H, Takita M, Matsumoto S, Yagishita A, Owada S, Esumi H, Tsuchihara K (2014). Epidermal growth factor receptor (EGFR) signaling regulates global metabolic pathways in EGFR-mutated lung adenocarcinoma. J Biol Chem.

[23] Tricker EM, Xu C, Uddin S, Capelletti M, Ercan D, Ogino A, Pratilas CA, Rosen N, Gray NS, Wong KK, Janne PA (2015). Combined EGFR/MEK Inhibition Prevents the Emergence of Resistance in EGFR-Mutant Lung Cancer. Cancer Discov.

[24] Eberlein CA, Stetson D, Markovets AA, Al-Kadhimi KJ, Lai Z, Fisher PR, Meador CB, Spitzler P, Ichihara E, Ross SJ, Ahdesmaki MJ, Ahmed A, Ratcliffe LE (2015). Acquired Resistance to the Mutant-Selective EGFR Inhibitor AZD9291 Is Associated with Increased Dependence on RAS Signaling in Preclinical Models. Cancer Res.

[25] Hui ST, Andres AM, Miller AK, Spann NJ, Potter DW, Post NM, Chen AZ, Sachithanantham S, Jung DY, Kim JK, Davis RA (2008). Txnip balances metabolic and growth signaling via PTEN disulfide reduction. Proc Natl Acad Sci USA.

[26] Ellinghaus P, Heisler I, Unterschemmann K, Haerter M, Beck H, Greschat S, Ehrmann A, Summer H, Flamme I, Oehme F, Thierauch K, Michels M, Hess-Stumpp H (2013). BAY 87–2243, a highly potent and selective inhibitor of hypoxia-induced gene activation has antitumor activities by inhibition of mitochondrial complex I. Cancer Med.

[27] Walter AO, Sjin RT, Haringsma HJ, Ohashi K, Sun J, Lee K, Dubrovskiy A, Labenski M, Zhu Z, Wang Z, Sheets M, St Martin T, Karp R (2013). Discovery of a mutant-selective covalent inhibitor of EGFR that overcomes T790M-mediated resistance in NSCLC. Cancer Discov.

[28] Foretz M, Guigas B, Bertrand L, Pollak M, Viollet B (2014). Metformin: from mechanisms of action to therapies. Cell Metab.

[29] Xiao B, Sanders MJ, Carmena D, Bright NJ, Haire LF, Underwood E, Patel BR, Heath RB, Walker PA, Hallen S, Giordanetto F, Martin SR, Carling D (2013). Structural basis of AMPK regulation by small molecule activators. Nat Commun.

[30] Ramalingam S, Yang JC, Lee CK, Kurata T, Kim DW, John T, Nogami N, Ohe Y, Janne PA (2016). LBA1_PR: Osimertinib as first-line treatment for EGFR mutation-positive advanced NSCLC: updated efficacy and safety results from two Phase I expansion cohorts. J Thorac Oncol.

[31] Engelman JA, Zejnullahu K, Mitsudomi T, Song Y, Hyland C, Park JO, Lindeman N, Gale CM, Zhao X, Christensen J, Kosaka T, Holmes AJ, Rogers AM (2007). MET amplification leads to gefitinib resistance in lung cancer by activating ERBB3 signaling. Science.

[32] Takezawa K, Pirazzoli V, Arcila ME, Nebhan CA, Song X, de Stanchina E, Ohashi K, Janjigian YY, Spitzler PJ, Melnick MA, Riely GJ, Kris MG, Miller VA (2012). HER2 amplification: a potential mechanism of acquired resistance to EGFR inhibition in EGFR-mutant lung cancers that lack the second-site EGFRT790M mutation. Cancer Discov.

[33] Planchard D, Loriot Y, Andre F, Gobert A, Auger N, Lacroix L, Soria JC (2015). EGFR-independent mechanisms of acquired resistance to AZD9291 in EGFR T790M-positive NSCLC patients. Ann Oncol.

[34] Wang DS, Jonker JW, Kato Y, Kusuhara H, Schinkel AH, Sugiyama Y (2002). Involvement of organic cation transporter 1 in hepatic and intestinal distribution of metformin. J Pharmacol Exp Ther.

[35] Segal ED, Yasmeen A, Beauchamp MC, Rosenblatt J, Pollak M, Gotlieb WH (2011). Relevance of the OCT1 transporter to the antineoplastic effect of biguanides. Biochem Biophys Res Commun.

[36] Crofford OB (1995). Metformin. N Engl J Med.

[37] Shah RR, Evans DA, Oates NS, Idle JR, Smith RL (1985). The genetic control of phenformin 4-hydroxylation. J Med Genet.

[38] Yuan P, Ito K, Perez-Lorenzo R, Del Guzzo C, Lee JH, Shen CH, Bosenberg MW, McMahon M, Cantley LC, Zheng B (2013). Phenformin enhances the therapeutic benefit of BRAF(V600E) inhibition in melanoma. Proc Natl Acad Sci USA.

[39] Schockel L, Glasauer A, Basit F, Bitschar K, Truong H, Erdmann G, Algire C, Hagebarth A, Willems PH, Kopitz C, Koopman WJ, Heroult M (2015). Targeting mitochondrial complex I using BAY 87-2243 reduces melanoma tumor growth. Cancer Metab.

[40] Baenke F, Chaneton B, Smith M, Van Den Broek N, Hogan K, Tang H, Viros A, Martin M, Galbraith L, Girotti MR, Dhomen N, Gottlieb E, Marais R (2016). Resistance to BRAF inhibitors induces glutamine dependency in melanoma cells. Mol Oncol.

[41] De Rosa V, Iommelli F, Monti M, Fonti R, Votta G, Stoppelli MP, Del Vecchio S (2015). Reversal of Warburg Effect and Reactivation of Oxidative Phosphorylation by Differential Inhibition of EGFR Signaling Pathways in Non-Small Cell Lung Cancer. Clin Cancer Res.

