# Supplementary material for: Inhibition of oxidative phosphorylation suppresses the development of osimertinib resistance in a preclinical model of EGFR-driven lung adenocarcinoma
Source: Oncotarget. 2016 Nov 16;7(52):86313–25. doi: 10.18632/oncotarget.13388 (PMC5349916; doi:10.18632/oncotarget.13388)
Supplement: Supplementary file 1 [file oncotarget-07-86313-s001.pdf]

Inhibition of oxidative phosphorylation suppresses the development of osimertinib resistance in a preclinical model of EGFR-driven lung adenocarcinoma

Supplementary Materials

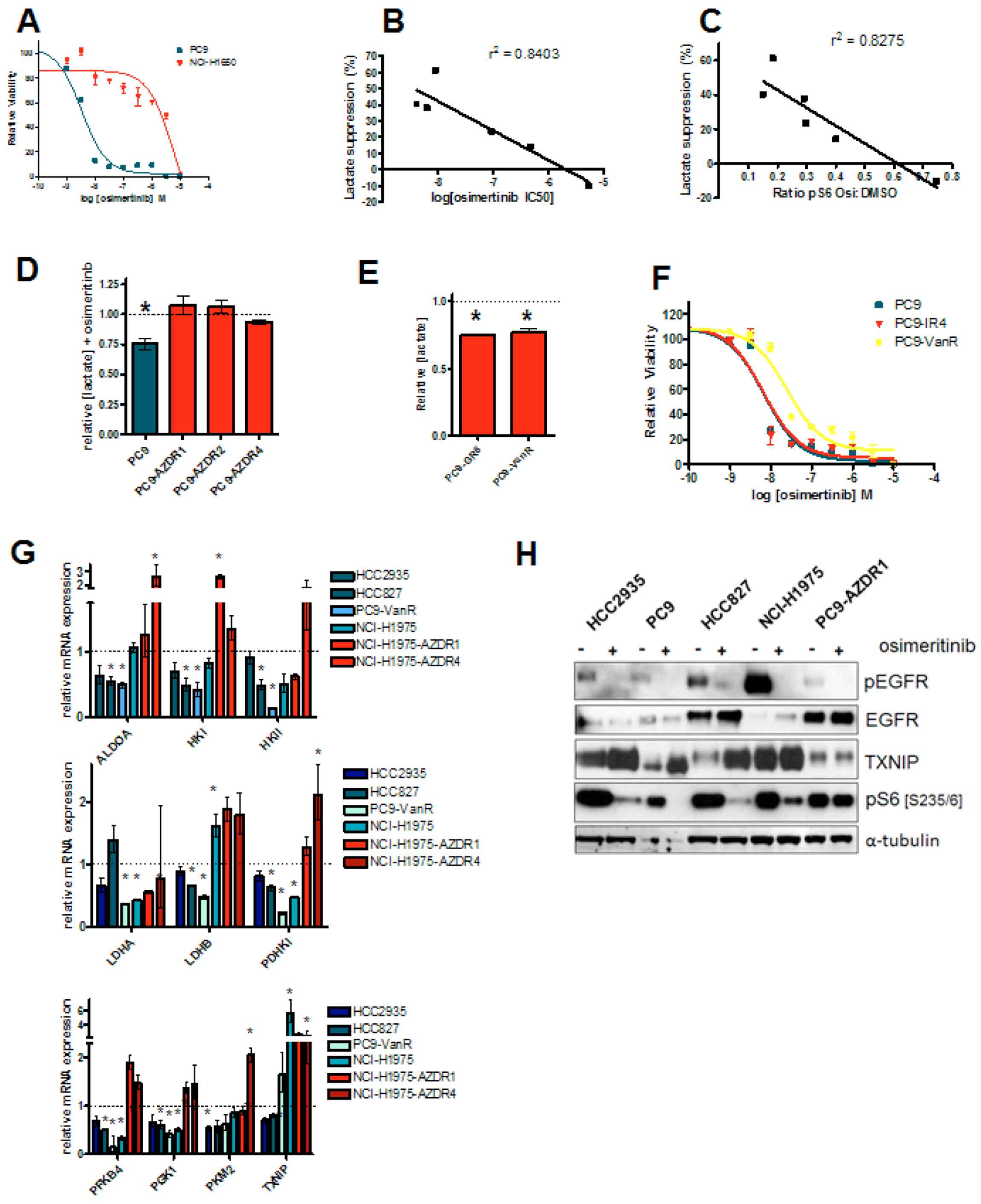

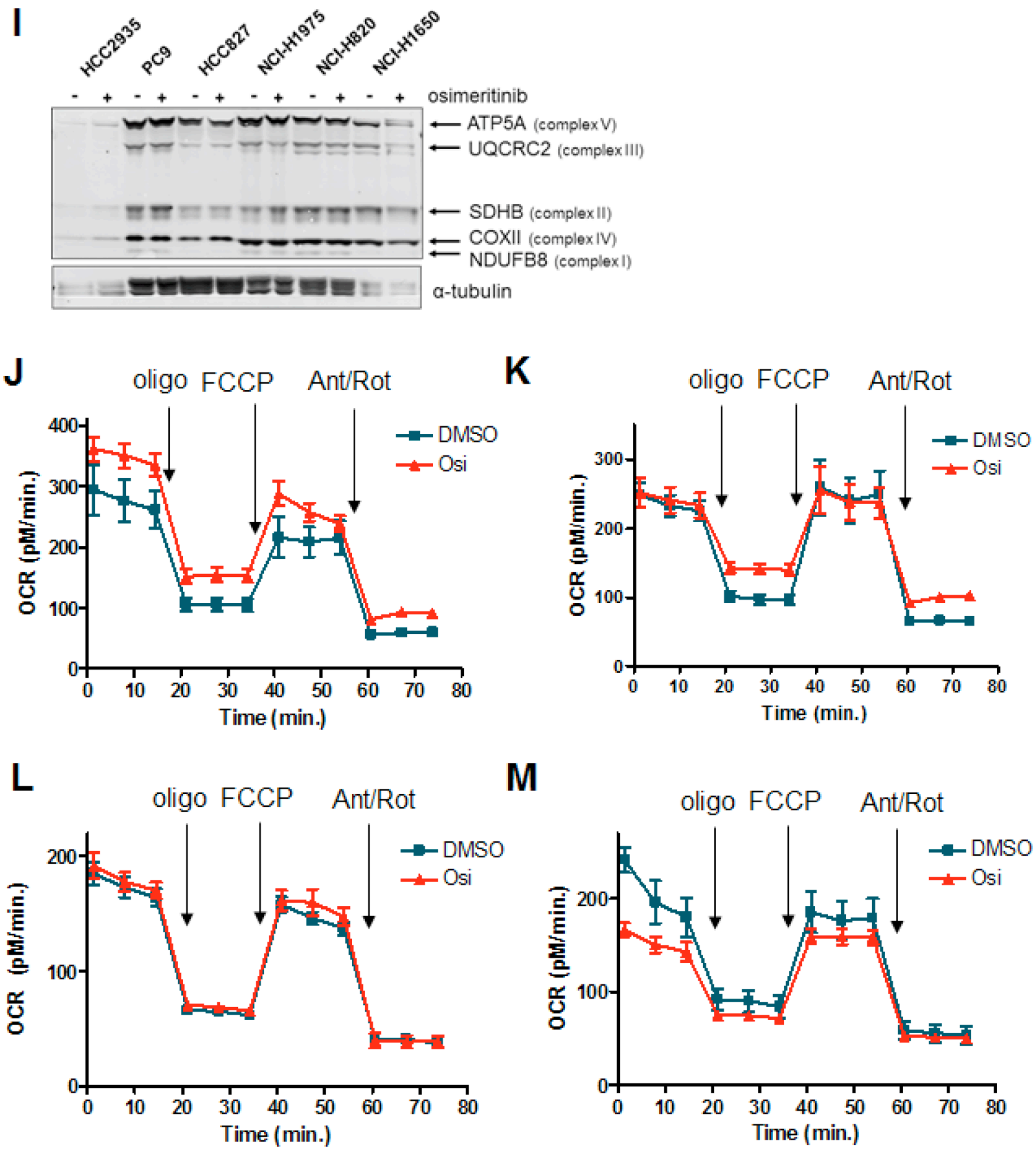

Supplementary Figure S1: (**A**) 96 h osimertinib growth response curve for PC9 and NCI-H1650 cell lines. (**B**) Plot of average osimertinib IC50 (log scale) vs. % lactate suppression for the panel of 6 cell lines described in figure 1C. (**C**) Plot of S6 phosphorylation after osimertinib treatment vs. % lactate suppression for the panel of 6 cell lines described in figure 1C. (**D**), (**E**) The indicated cell lines were treated for 24 h with 160 nM osimertinib and vehicle control, and conditioned media was analysed for lactate concentration. Values shown are means relative to vehicle control +/− SEM (*$p < 0.05$; $n = 3$). (**F**) 96 h osimertinib growth response curve for PC9, PC9-GR6 and PC9-VanR cells. (**G**) The indicated cells were treated with 160 nM osimertinib or vehicle control for 24 h, RNA was isolated and relative levels of mRNA expression was determined by qPCR. Values shown are means relative to vehicle control +/− SEM ($n = 3$). (**H**, **I**) Cell lysates were subjected to Western blotting for the indicated proteins. (**J**) PC9 (**K**) NCI-H1975 (**L**) PC9-AZDR1 and (**M**) NCI-H1975-AZDR1 cells were plated in 96 well plates, treated for 24 h with osimertinib (160 nM for PC9/PC9-AZDR1; 300 nM for NCI-H1975/NCI-H1975-AZDR1), and analysed for oxygen consumption using the Seahorse Cell Mito Stress Test as described in the Materials and Methods.

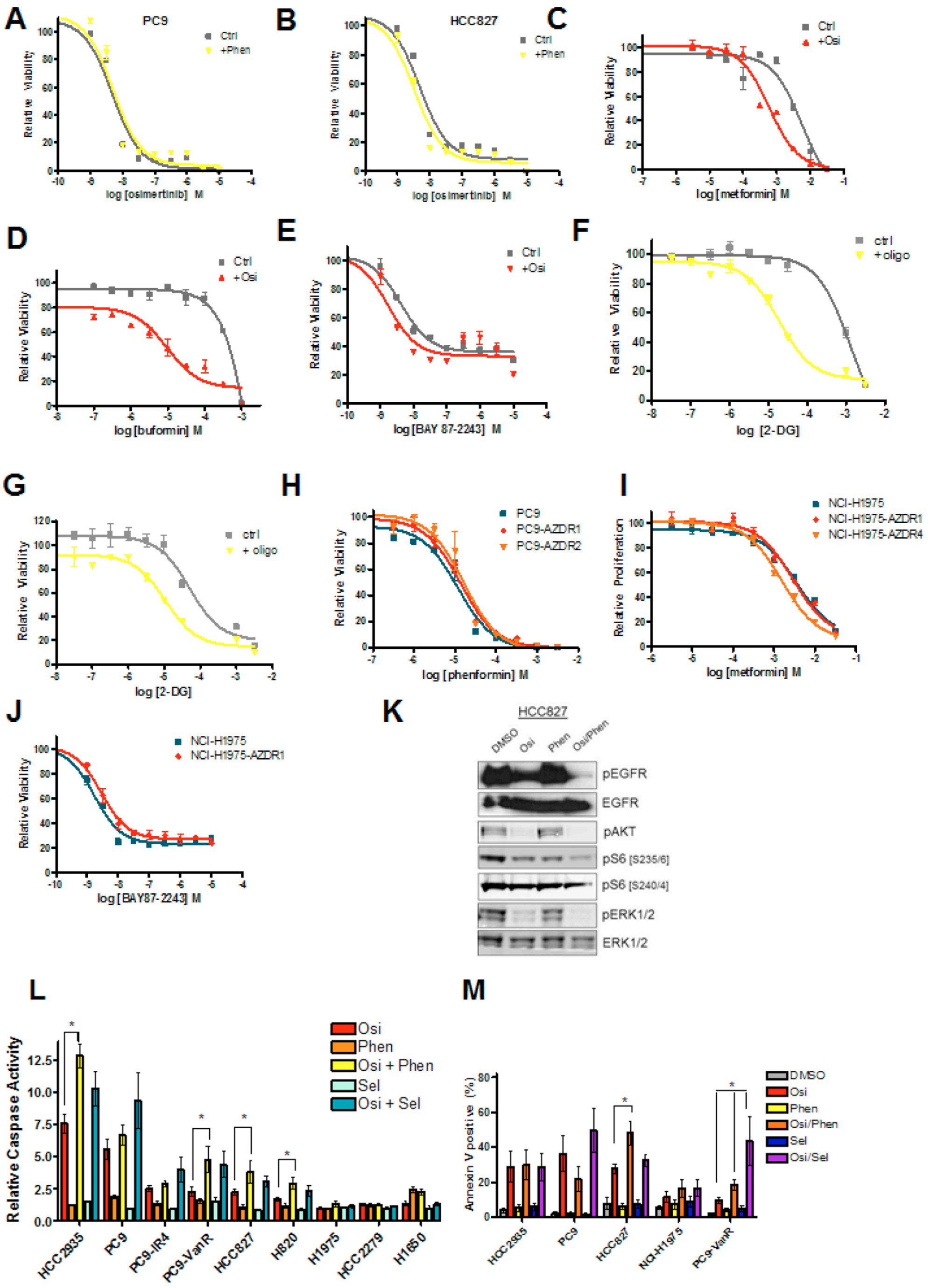

Supplementary Figure S2: (**A**) 96 h osimertinib growth curve for PC9 cells in the presence or absence of 30 µM phenformin. (**B**) 96 h osimertinib growth curve for HCC827 cells in the presence or absence of 100 µM phenformin. (**C**) 96 h metformin growth curve for NCI-H1975 cells in the presence or absence of 160 nM osimertinib. (**D**) 96 h buformin growth curve for NCI-H1975 cells in the presence or absence of 160 nM osimertinib. (**E**) 96 h BAY-87-2243 growth curve for NCI-H1975 cells in the presence or absence of 160 nM osimertinib. (**F**) PC9 and (**G**) NCI-H1975 cells: 96 h growth response curve of NCI-H1975 cells to 2-deoxyglucose (2-DG) in the presence or absence of 100 nM oligomycin. (**H**) 96 h growth response curve of PC9, PC9-AZDR1 and PC9-AZDR2 cells to phenformin. (**I**) 96 h growth response cureve of NCI-H1975, NCI-H1975-AZDR1 and NCI-H1975-AZDZR4 cells to metformin. (**J**) 96 h growth response curve of NCI-H1975, and NCI-H1975-AZDZR4 cells to BAY-87-2243. (**K**) Cells were treated for 24 h with either osimertinib (160 nM), phenformin (0.1 mM) or the combination of both. Cells were lysed and subjected to Western blotting for the indicated proteins. (**L**) Data corresponding to that of Figure 2E, but including phenformin and selumetinib monotherapy groups. Cells were treated for 48 h with the indicated compounds and subjected to a caspase 3/7 assay. Readings were normalized to cell viabiilty as measured by Cell Titer Glo, and values shown are means relative to vehicle control +/− SEM ($n = 3$). (**M**) Cells were treated for 48 h with the indicated compounds, stained with Annexin V and scored using a fluorscent microscope as described in the Materials and Methods. Values shown are % Annexin V-positive cells as a proportion of overall number of cells.

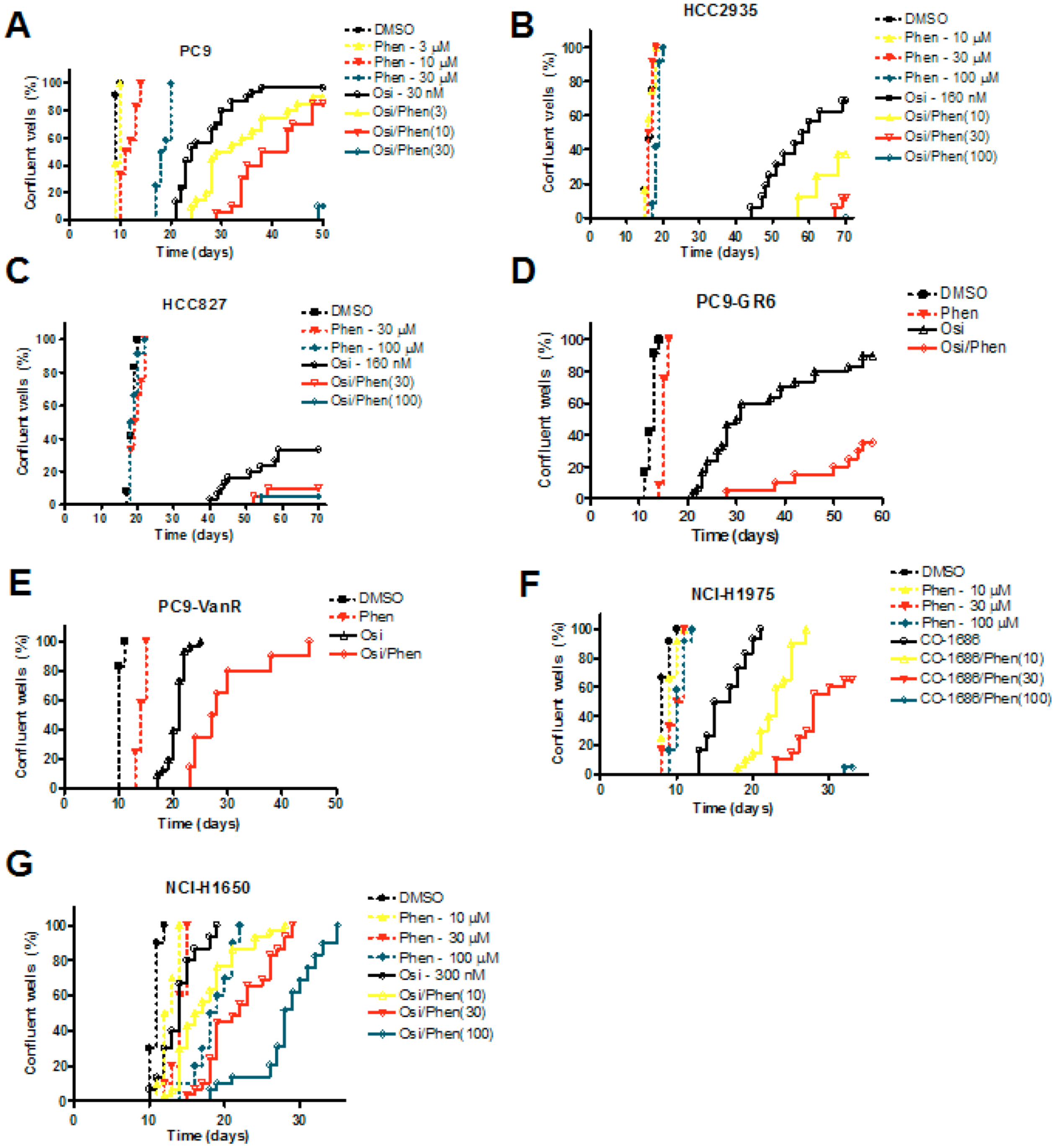

Supplementary Figure S3: (**A–G**) Cells were plated at low density, treated with the indicated compounds and scored for resistance as described in the materials and methods.

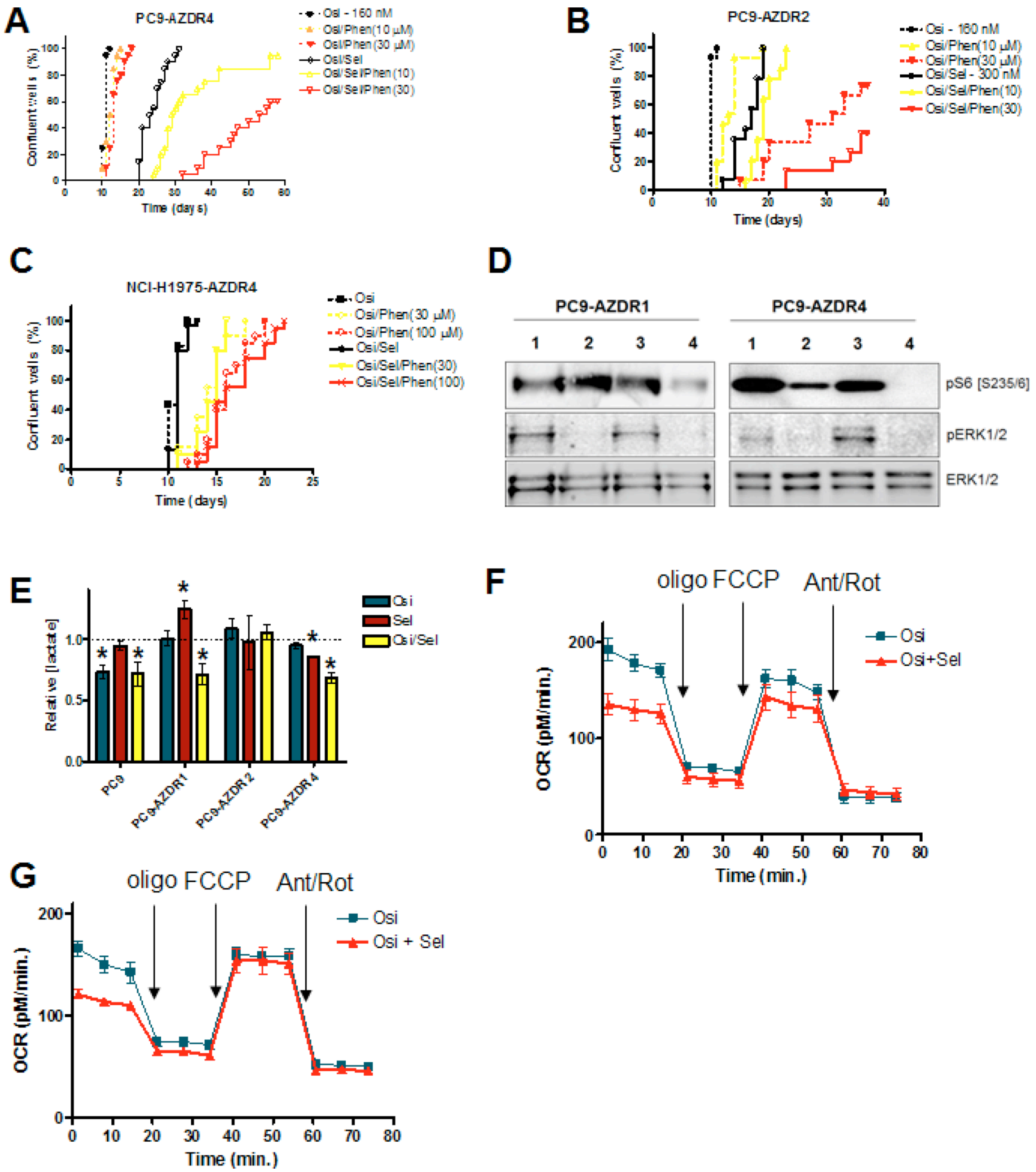

Supplementary Figure S4: (**A**) PC9-AZDR4, (**B**) PC9-AZDR2 and (**C**) NCI-H1975-AZDR4 cells were plated at low density, treated with the indicated compounds and scored for resistance as described in the Materials and Methods. (**D**) PC9-AZDR1 and PC9-AZDR4 cells were treated with either 1. Osimertinib (160 nM) 2. Osimertinib + selumetinib (300 nM) 3. Osimertinib + phenformin (30 µM) or 4. Osimertinib + selumetinib + phenformin for 24 h, lysed and subjected to Western blotting for the indicated proteins. (**E**) The indicated cell lines were treated for 24 h with osimertinib (160 nM), selumetinib (300 nM), osimertinib + selumetinib or vehicle control, and conditioned media was analysed for lactate concentration. Values shown are means relative to vehicle control +/− SEM (*$p < 0.05$; $n = 3$). (**F**) PC9-AZDR1 and (**G**) NCI-H1975-AZDR1 cells were plated in 96 well plates, treated for 24 h with osimertinib alone (160 nM for PC9-AZDR1; 300 nM for NCI-H1975-AZDR1) or in combination with 300 nM selumetinib, and analysed for oxygen consumption using the Seahorse Cell Mito Stress Test as described in the Materials and Methods.

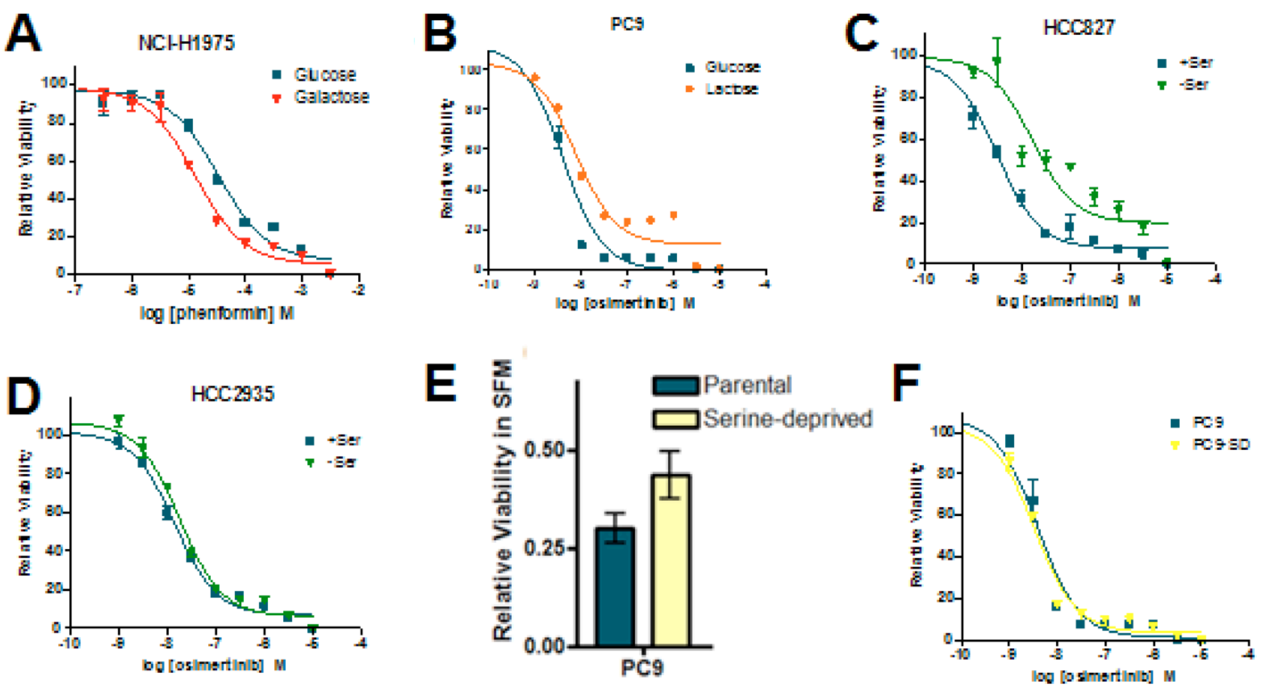

Supplementary Figure S5: (**A**) 96 h phenformin growth response curve for NCI-H1975 cells growth in DMEM containing 5 mM or either glucose or galactose. (**B**) 96 h osimertinib growth response curve for PC9 cells growth in DMEM containing 5 mM or either glucose or lactose. (**C**) 96 h osimertinib growth response curve for HCC827 cells grown in either SFM or SFM+SG. (**D**) 96 h osimertinib growth response curve for HCC2935 cells grown in either SFM or SFM+SG. (**E**) PC9and SD cells were growth in –Ser or +Ser for 96 h, and viability analysed by Cell Titer Glo. Values represent the mean viability in -Ser compared to +Ser (n = 5). (**F**) 96 h osimertinib growth response curve for PC9 cells growth in RPMI.

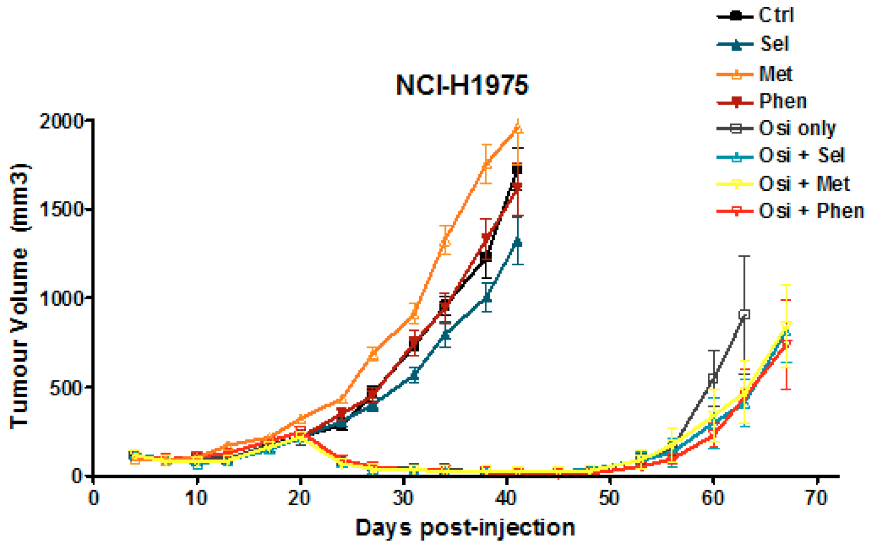

Supplementary Figure S6: 2 × 10^7 NCI-H1975 cells were implanted into the flank of nude (nu/nu) mice. When tumours were approximately 200 mm^3 mice were randomized into groups and treated by oral gavage daily with a) vehicle control (n = 10) b) 25 mg/kg selumetinib (n = 10) c) 300 mg/kg metfomrin (n = 10) d) 100 mg/kg phenformin (n = 10) e) 5 mg/kg osimertinib (n = 6) f) osimertinib + selumetinib (n = 6) g) osimertinib + metformin (n = 5) or h) osimertinib + phenformin (n = 6). Dosing was stopped after 28 days and tumours were monitored until one or more tumours from a group reached a size of 2000 mm^3 at which time all mice from that group were sacrificed. Values shown are means +/− SEM. These animal studies were conducted in accordance with AZ Global Bioethics Policy, the French & European Regulations and NRC Guidance on care and use of laboratory animals.

Supplementary Table S1: Lactate suppression in EGFR-mutant cell lines inversely correlates with lactate suppression after osimertinib treatment (see Figure 1C)

Cell Line	Average Osimertinib IC50 (nM)	Lactate Suppression (%)
HCC2935	8.9	61.0
PC9	4.1	40.2
HCC827	6.3	37.8
NCI-H1975	92.3	23.3
NCI-H820	487	14.0
NCI-H1650	5393	(−10.3)

Supplementary Table S2: Cell lines used in this study

Cell Line	Source	EGFR Mutation Status	Other Notable Mutations
HCC827	ATCC	Exon 19 del	-
HCC2935	ATCC	Exon 19 del	-
NCI-H460	ATCC	Wt	KRAS Q61H
NCI-H820	ATCC	Exon 19 del; T790M	-
NCI-H1650	ATCC	Exon 19 del	PTEN del
NCI-H1975	ATCC	L858R; T790M	-
NCI-H1975 AZD9291 resistant 1	In-house	L858R; T790M	-
NCI-H1975 AZD9291 resistant 4	In-house	L858R; T790M	NRAS Q61K
PC9	ECACC	Exon 19 del	-
PC9 AZD9291 resistant 1	In-house	Exon 19 del	NRAS gain
PC9 AZD9291 resistant 2	In-house	Exon 19 del	NRAS G12V
PC9 AZD9291 resistant 4	In-house	Exon 19 del	-
PC9 Gefitinib-resistant 6	In-house	Exon 19 del; T790M	-
PC9 Vandetinib-resistant	In house	Exon 19 del; T790M	-

Supplementary Table S3: Commercially purchased antibodies used in this study

Antibody Target	Source	Catalogue number	Dilution	Species	Detection method
pAKT (S473)	Cell Signaling	4060	1:1000	Rabbit	Film
EGFR	Cell Signaling	2232	1:1000	Rabbit	Film
pEGFR (Y1068)	Cell Signaling	2234	1:1000	Rabbit	Film
ERK1/2	Cell Signaling	4696	1:2000	Rabbit	LICOR
pERK1/2	Cell Signaling	4370	1:2000	Mouse	LICOR
Mitochondrial ETC	Abcam	ab110411	1:2000	Mouse	LICOR
pPKM2 (Y105)	Cell Signaling	3827	1:1000	Rabbit	LICOR
pS6 (S235/6)	Cell Signaling	4858	1:2000	Rabbit	LICOR
pS6 (S240/4)	Cell Signaling	5364	1:2000	Rabbit	LICOR
α-tubulin	Sigma	T6199	1:5000	Mouse	LICOR
